# Endoscopic management of impacted basket in the pancreatic duct

**DOI:** 10.1055/a-2819-2846

**Published:** 2026-04-02

**Authors:** Khalid Ahmed, Guru Trikudanathan

**Affiliations:** 1 Department of Medicine, Division of Gastroenterology, Hepatology and Nutrition, University of Minnesota, Minneapolis, Minnesota, United States

Methods for extracting pancreatic duct (PD) stones include pancreatic sphincterotomy, lithotripsy, and retrieval with balloons or baskets 1 . Basket impaction in the common bile duct is well recognized, impaction within the PD is uncommon 2 3 . We present a case of an impacted basket during the attempted removal of a PD stone and describe a conservative approach that avoided surgical intervention.

**Fig. 1:**
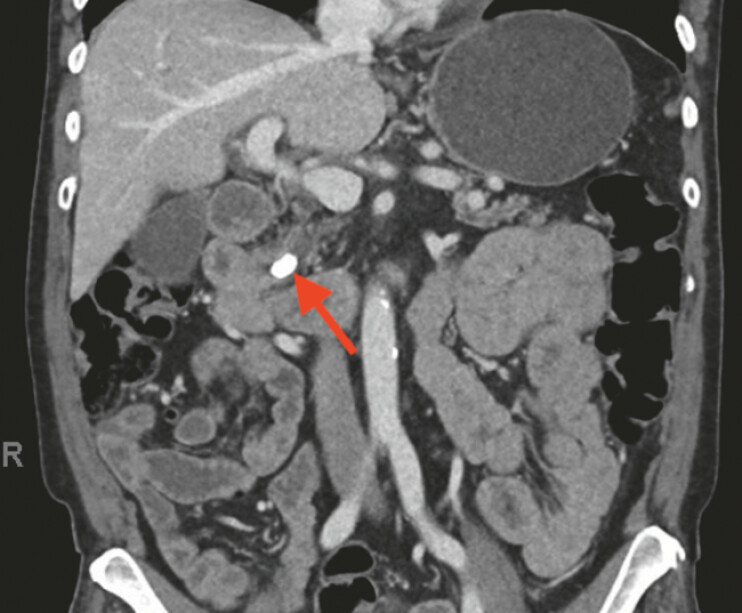
Computed topography showed the pancreatic duct stone.

**Fig. 2:**
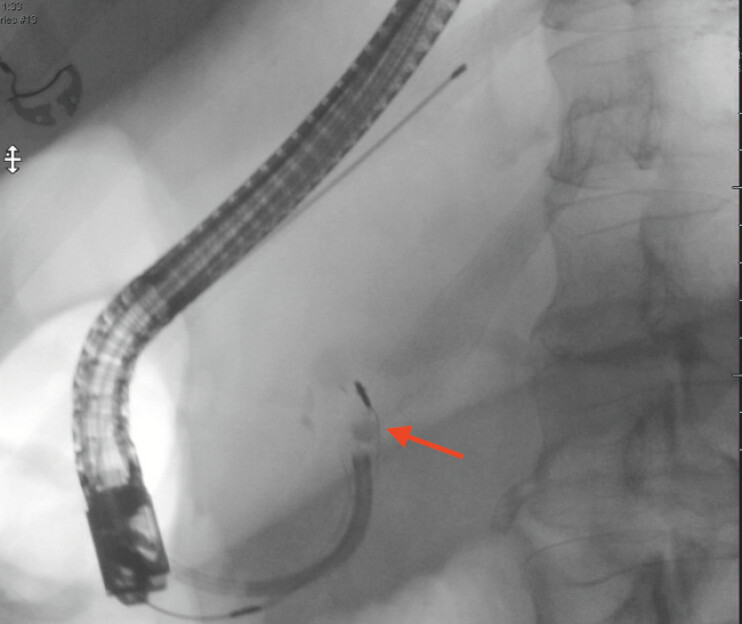
Fluoroscopy showing the stone and impacted basket.

A 74-year-old woman with diabetes, hyperlipidemia, hypertension, and chronic kidney disease (stage 3) was evaluated for persistent abdominal pain. Computed topography (CT) revealed a large obstructing stones in the PD ( Fig. 1 ). She previously underwent two sessions of extracorporeal shockwave lithotripsy (ESWL) with 5,000 shocks each, resulting in only minimal symptom improvement. A tandem endoscopic retrograde cholangiopancreatography (ERCP) was performed with the second session of ESWL, and cannulation of the ventral PD revealed. A 6-mm pancreatic sphincterotomy and 4-mm balloon dilation were completed. During stone extraction, a flower basket became impacted within the PD and could not be withdrawn ( Fig. 2 ). Attempts with an emergency electrohydraulic lithotripsy (EHL) were unsuccessful.

Pancreatoscopy was then used to directly visualize the stone. Additional EHL resulted in partial fragmentation, allowing the basket and stone to be grasped with jumbo forceps; however, both became lodged at the ampulla. Following the dilation of the PD orifice to 6 mm, the stone and basket were successfully removed. Two PD stents were placed, and no bleeding, leak, or perforation was noted ( Video 1 ). At a 6-week follow-up of ERCP, the pancreatogram confirmed complete stone clearance, prior stents were removed, and a new stent was placed. Four-week radiography confirmed spontaneous stent passage, and the patient reported significant improvement in pain.

Pancreatoscopy with electrohydraulic lithotripsy (EHL).Video 1

Basket impaction within the PD is a challenging complication with limited management guidance. This case highlights a stepwise, minimally invasive strategy that achieved safe removal without surgery.

Endoscopy_UCTN_Code_CPL_1AK_2AF
